# Proprotein Convertase Furin Regulates Apoptosis and Proliferation of Granulosa Cells in the Rat Ovary

**DOI:** 10.1371/journal.pone.0050479

**Published:** 2013-02-13

**Authors:** Xiaokui Yang, Qingxin Wang, Zhiying Gao, Zhi Zhou, Sha Peng, Wen-Lin Chang, Hai-Yan Lin, Weiyuan Zhang, Hongmei Wang

**Affiliations:** 1 Department of Human Reproductive Medicine, Beijing Obstetrics and Gynecology Hospital, Capital Medical University, Beijing, China; 2 State Key Laboratory of Reproductive Biology, Institute of Zoology, Chinese Academy of Sciences, Beijing, China; 3 Department of Obstetrics and Gynecology, PLA General Hospital, Beijing, China; 4 Graduate School of the Chinese Academy of Sciences, Beijing, China; Fudan University, China

## Abstract

Folliculogenesis is tightly controlled by a series of hormones, growth factors and cytokines, many of which are secreted as proproteins and require processing by proteases before becoming functional. Furin is a member of the subtilisin-like proteases that activate large numbers of proprotein substrates and is ubiquitously expressed and implicated in many physiological and pathological processes. However, the precise role of furin during folliculogenesis has not been thoroughly investigated. The goal of the present work is to identify the role of furin in the development of granulosa cells during folliculogenesis, using immunohistochemistry, RT-PCR, Western blot and functional studies in primary cultured rat granulosa cells. Our results demonstrate that furin is highly expressed in granulosa cells and oocytes of the ovary with very limited expression in other ovarian cells such as the epithelial, stromal or theca cells. Furin siRNA significantly increases apoptosis of the granulosa cells from large antral/preovulatory follicles, in part via downregulation of the anti-apoptotic proteins, XIAP and p-AKT. On the contrary, furin siRNA markedly decreases proliferation of granulosa cells based on the downregulation of proliferation cell nuclear antigen (PCNA). Taken together, these data suggest that furin may play an important role in regulating apoptosis and proliferation of granulosa cells.

## Introduction

Folliculogenesis is crucial for female reproductive function. During this process, atresia occurs in most follicles and only a few follicles mature and ovulate [1], [2], [3], [4]. This process is characterized by transformation of granulosa cells from a flattened to a cuboidal shape, and by their proliferation, recruitment and differentiation, which is tightly controlled in a temporal and spatial manner and changes in oocyte size and morphology that reflect both nuclear and cytoplasmic maturation [5], [6]. Folliculogenesis is tightly controlled by a series of paracrine and endocrine factors, hormones, growth factors and cytokines, many of which are secreted as proproteins and require processing by proteases before becoming functional. Abnormality of this process will result in follicular dysplasia which is related to Premature Ovarian Failure (POF) and Polycystic Ovary Syndrome (PCOS) that seriously threaten the health and reproduction of women [7], [8], [9].

Proprotein convertase subtilisin/kexin-like proteases (PCSKs) belong to the family of serine proteases of the subtilisin subtype. Their main function is to activate large numbers of proprotein substrates. Based on structural similarities to yeast and bacterial proteases, PCSKs are further subdivided into kexin-like clans, including PCSK1 (PC1, PC3, SPC3), PCSK2 (PC2, SPC2), PCSK3 (Furin, PACE, SPC1), PCSK4 (PC4, SPC5), PCSK5 (PC5, PC6, SPC6), PCSK6 (PACE4, SPC4) and PCSK7 (PC7, PC8, LPC, SPC7) detected in the mammalian cells, pyrolysin-like clans include PCSK8 (SKI-1, S1P) or proteinase K-like clans include PCSK9 (NARC-1) [10], [11], [12], [13]. PCSK1 and PCSK2 are expressed specially in the neural and endocrine tissues, while PCSK4 is in the reproductive organs and the placenta. Furin, PCSK5, PCSK6 and PCSK7 are extensively expressed in many tissues [14]. The activity of many proteins is regulated by PCSKs, including neural peptide, peptide hormones, growth factors and their receptors and the family of matrix metalloproteinases (MMPs) [14].

Furin, which was found the earliest and studied the most extensively [10], [15], plays a crucial role in a variety of physiological processes and was involved in the pathology of many diseases [16]. It has been reported that furin was highly expressed in tumor tissues and placentas. Furin has also been shown to promote the metastasis of many types of cancer cells [17], [18], [19], [20]. Moreover, our previous work showed that furin was highly expressed in the extravillous trophoblast cells at early stage of pregnancy and promoted invasion and migration of extravillous trophoblast cells [21]. There are very few reports regarding the possible role of furin in the development of granulosa cells during folliculogenesis. Tomohiro S and colleagues [22] demonstrated that newly synthesized Zona Pellucida C (proZPC) is cleaved by furin to generate mature ZPC prior to secretion, while Brian and colleagues [23] reported that furin participates in ovulation via both the cAMP and epidermal growth factor receptor (EGFR) pathways. Furthermore, the highest expression of furin mRNA occurs in oocytes of small growing follicles in medaka, Oryzias latipes [24]. A recent study by Antenos and colleagues also showed that furin mRNA is inversely related to the inhibin subunits and is significantly decreased during follicular development in the developing mouse ovaries [25].

In the present study, our goal was to identify the function of furin during folliculogenesis. To that end, the expression of furin in the follicles at different stages of development was studied using immunohistochemistry, and the role of furin in the proliferation and apoptosis of granulosa cells was investigated in primary culture of rat granulosa cells. We found that furin is highly expressed in granulosa cells and oocytes of the ovary compare to other ovarian cells such as the epithelial, stromal or theca cells. Furthermore, furin siRNA significantly increases apoptosis and decreases proliferation of granulosa cells. The results suggest that furin does participate in apoptosis and proliferation of granulosa cells during folliculogenesis.

## Materials and Methods

### Animal preparation

Twenty-one-day immature Sprague Dawley rats were purchased from the animal center of Peking University Health Science Center and maintained on a 12-h light, 12-h dark cycles and given food and water ad libitum. All procedures were carried out in accordance with the Guidelines for the Care and Use of Laboratory Animals, and were approved by the Ethics Committee of Institute of Zoology, Chinese Academy of Sciences. To obtain follicles at different stages of development for immunohistochemistry, rats were injected s. c. with PMSG (10 IU) for 0 (n = 5), 24 (n = 5), and 48 h (n = 5), and ovaries were collected and fixed in 4% paraformaldehyde (PFA), then dehydrated through a graded series of ethanol before being processed for paraffin-embedding and sectioning.

### Immunohistochemistry

Immunohistochemistry (IHC) was performed with a biotin-streptavidin-peroxidase (SP) and diaminobenzidine (DAB, Zhongshan Golden Bridge Corp, Beijing, China) as Zhou et al described [21]. In brief, five-micrometer sections from paraffin-embedded ovaries were deparaffinized in xylene and rehydrated through a graded series of ethanol. Antigen retrieval was achieved by subjecting the sections to microwave irradiation (91–94°C) for 15 min in solution of retrieval (1.8 mM citric acid and 8.2 mM sodium citrate) and cooled down in the room temperature. Sections were then sequentially incubated with 3% H_2_O_2_ for 10 min to eliminate endogenous hydrogen peroxidase activity, blocking serum for 20 min in the dark, primary antibody furin (ab3467, Abcam, Cambridge, UK; diluted 1∶600 in PBS) overnight at 4°C (for negative control, blocking serum instead), secondary rabbit-antibody for 30 min (37°C), and horseradish peroxidase-streptavidin for 15 min before visualization with DAB. Finally, sections were mounted for photography. Photomicrographs were taken with a Nikon digital camera (Nikon, Tokyo, Japan) using SPOT Advanced Plus software (Diagnostic, America).

### Primary culture of rat granulosa cells

As previously described [26], twenty-one-day immature Sprague Dawley rats were injected s. c. with PMSG (10 IU) for 48 h, and ovaries were collected. Granulosa cells from large antral/preovulatory follicles were harvested by follicular puncture with a 26.5-gauge needle. Cell clumps and oocytes were removed by filtering the cell suspensions through a 40-μm nylon cell strainer. Cells were collected by centrifuging the cell suspension at 800 rpm. The number of viable granulosa cells was determined by the trypan blue dye-exclusion test. Cells were plated in 60-mm dishes or 24-well plates at 30% confluence in RPMI 1640 medium (Gibco, MA) with 10% fetal bovine serum under a humidified atmosphere of 95% air and 5% CO_2_. For cell death detection and Western blotting (two 60-mm dishes), one rat was used. For cell proliferation assay (24-well plates), one rat was used.

### RNA interference

Granulosa cells were transfected with 100 nM furin siRNA (5′- CACGGAUGAUCUCCACAUCAUUCUC -3′; Invitrogen, MD; Genbank ID for furin: NM_019331) or control siRNA (scrambled siRNA, a universal negative control; Invitrogen) with Lipofectamine 2000 (Invitrogen), according to the manufacturer's instructions. FITC labeled positive control siRNA (Invitrogen) was included to show the efficiency of transfection, which was above 90%.

### Cell death detection

Granulosa cells were cultured on the coverslip, and infected with furin siRNA or control siRNA for 48–72 h, as previously described [27], [28]. The coverslip was washed 3 times with PBS, fixed in 4% PFA for 10 min and incubated with Hoechst 33258 staining buffer (12.5 μg/ml; Sigma-Aldrich, St Louis, MO) for 5 min. After several washes, the apoptotic cells were identified morphologically by fluorescent microscopy (Nikon, Tokyo, Japan) equipped with a UV filter. Healthy cells show round and intact nuclei, whereas apoptotic cells exhibit morphological changes with karyopyknosis or fragmentation. At least 300 cells in a selected area were counted in each treatment group. Cells were counted blinded to sample identity to avoid experimental bias. Three independent replicate experiments were performed.

### Protein extraction and Western blot

Western blot analysis was performed as previously described [29] on the cells not used for assesment of apoptosis. In summary, whole cell proteins were extracted with whole cell lysis buffer (4 mM EGTA, 3 mM EDTA, pH 8.0, 0.5% NP40, 12.5 mM HEPES, 1 mM DTT 0.5 mM Na_3_VO_4_, 125 mM NaF, 2.5 mg/ml aprotinin, 25 mg/ml Trypsin inhibitor, 25 mM PMSF). Proteins were subjected to SDS-PAGE. Separated proteins were transferred electrophoretically onto a nitrocellulose membrane (Pall Corporation, Pensacola, FL). After being blocked with 5% skim milk for 1 h, the membrane was sequentially incubated with primary antibodies (furin, X-linked Inhibitor of Apoptosis Protein [XIAP; Mab822, R&D, Minneapolis, MN], cleaved caspase-3 [sc-7148, Santa Cruz, CA], p-Akt [Thr 308; sc-16646-R, Santa Cruz, CA], PTEN [Mab847, R&D, Minneapolis, MN], PCNA [ZM0213, Zymed Lab Inc., CA]) or α-tubulin [ab7291, Abcam, Cambridge, UK; a loading control] overnight at 4°C, and horseradish peroxidase-conjugated secondary antibodies (Zhongshan Golden Bridge Corp, Beijing, China) for 2 h. Signals were developed using the Enhanced Chemiluminescence System (Pierce, Rockford, IL).

### RNA isolation and reverse transcription-PCR

Total RNA was isolated from rat granulosa cells using TRIzol reagent according to the manufacturer's instructions (Invitrogen Inc, Carlsbad, CA). Briefly, total RNA (2 μg) was used for cDNA synthesis by reverse transcription. The cDNAs obtained were amplified by using specific primers as follows [23]: Furin (597 bp): 5′- ATCTCAACGCTAACGATTGG -3′(sense), 5′- AGAAACCTTCCTCACACACC -3′ (antisense); Rpl32 (138 bp): 5′-GAAGCCCAAGATCGTCAAAA-3′ (sense), 5′- AGGATCTGGCCCTTGA ATCT-3′(antisense).

To detect the mRNA of furin, PCR was performed at 94°C for 5 minutes, followed by 30 cycles of 94°C for 30 seconds, 62°C for 45 seconds, and 72°C for 45 seconds, finally 72°C for 7 minutes. Rpl32 expression was used as an internal control. Three independent replicate experiments were performed.

### Cell proliferation assay

Granulosa cells were seeded in a 24-well plate at 30% confluence. Transfection of furin siRNA was performed with Lipofectamine 2000 as described above. For the MTT assay, the culture medium was changed to 500 µl RPMI 1640 medium without fetal bovine serum with 10% MTT reagent (3-(4,5-dimethylthiazol (-z-y1)-3,5-diphenyltetrazoliumbromide; Apllygen Corp. Beijing, China) for 4 h and 500 μl DTW (Triton X-100:DMF:H2O = 1∶3∶2) for 30 min before being transferred to a 96-well plate for analysis. The optical density of each well at 0, 24, 48 and 72 h was measured at 570 nm (Beckman DU530, Fullerton, CA). In parallel, trypsinized cells were analyzed at 0, 24, 48 and 72 h using the Scepter cell counter (Merck Millipore, America). The experiments were performed in triplicate.

### Statistical analysis

All data are represented as means ± SD. Statistical analysis was performed using a One-sample K-S test of SPSS11.5 for testing distribution and Independent-samples T test for testing differences. *, *P*<0.05; **, *P*<0.01.

## Results

### Furin was highly expressed in the granulosa cells of rat ovaries

**Figure 1:**
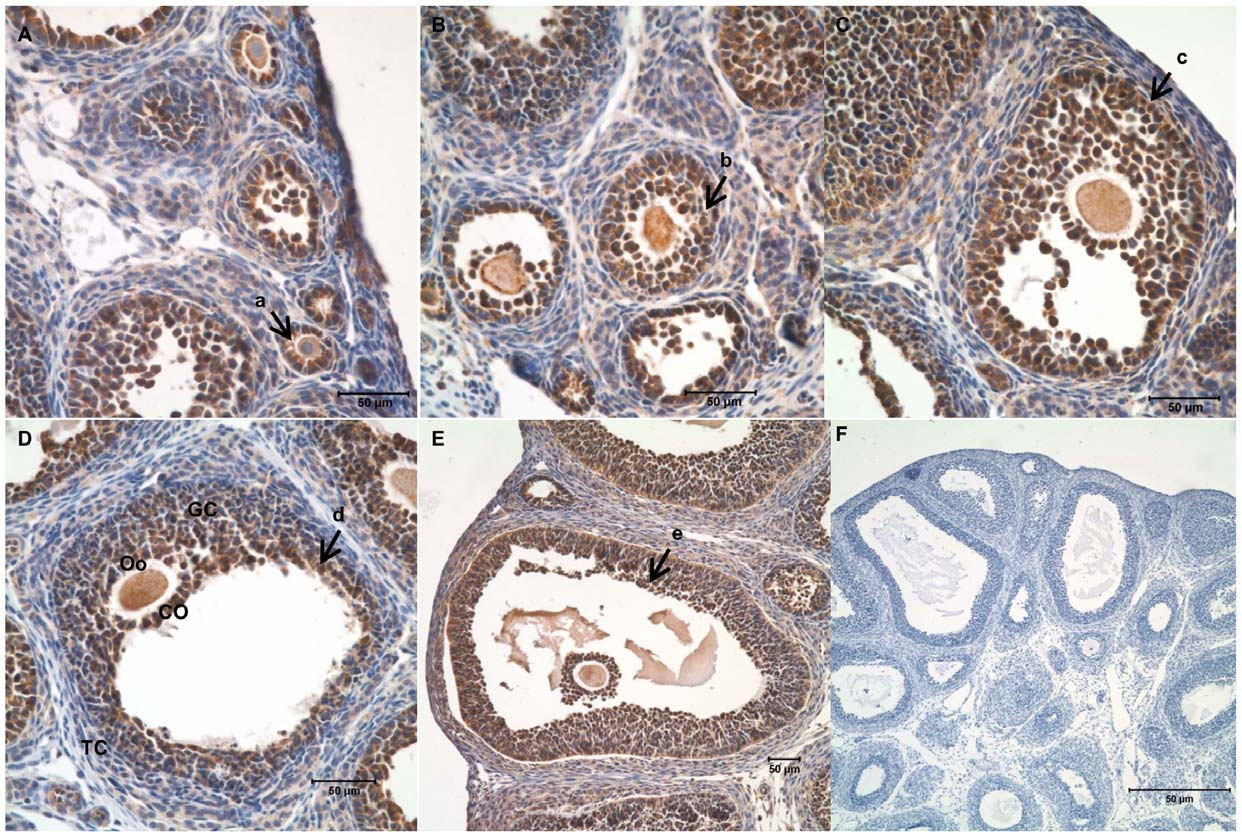
Expression of furin in follicles at different stages of folliculogenesis in the rat ovary. Ovaries from PMSG (10 IU; 0, 24, or 48 h)-primed immature rats were processed for paraffin section. Furin was immunolocalized by immumohistochemistry. Sections from the forty-eight hour ovary were included as the negative control and no positive signals were detected. A–E: Follicles at different stages of development, A–B: the section of 0 h after PMSG injection, C–D: the section of 24 h after PMSG injection, E–F: the section of 48 h after PMSG injection, a: primary follicle, b: pre-antral follicle, c: early antral follicle, d: antral follicle, e: mature follicle; GC: granulosa cell, TC: theca cell, Oo: oocyte, CO: cumulus oophorus. F: negative control. Bar = 50 μm.

**Figure 2:**
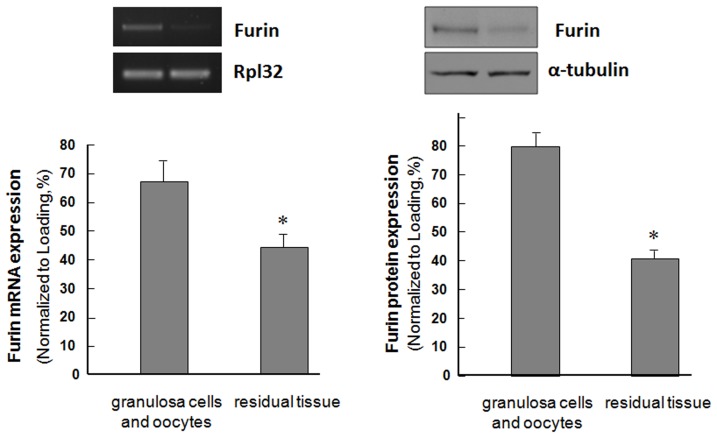
Expression of furin protein and mRNA level in the rat ovary. Furin protein and mRNA level in rat granulosa cells and oocytes and the other ovarian cells.

In order to detect the expression pattern of furin during folliculogenesis, immature rats were s. c. injected with pregnant mare serum gonadotropin (PMSG) for 0, 24, or 48 h. Harvested ovaries were sectioned and subjected to immunohistochemistry analysis. Results shown in Figure 1 indicated that furin was highly expressed in the granulosa cells and oocytes of primary follicles, pre-antral follicles, early antral follicles and antral follicles. There were no visible differences between the furin signals in the granulosa cells from follicles at different stages of development. The furin protein level in other ovarian cells such as the epithelial cells, stromal cells or theca cells was low to undectable. Similarly, furin mRNA was highly expressed in granulosa cells and oocyte, compared with other ovarian cells such as the epithelial cells, stromal cells or theca cells (Figure 2).

### Furin siRNA increased apoptosis of granulosa cells in rats

**Figure 3:**
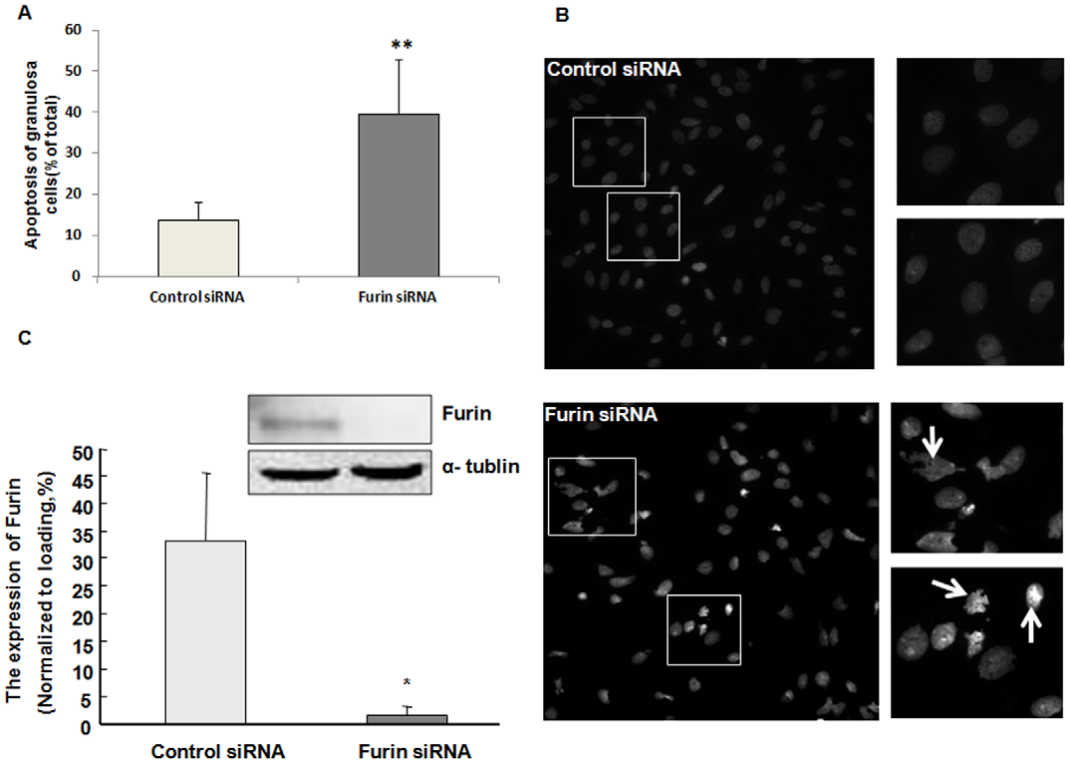
Furin siRNA significantly increased apoptosis of granulosa cells. Granulosa cells transfected with furin siRNA or control siRNA were incubated with Hoechst staining buffer. A. Cells were counted “blindly” three times. Granulosa cells transfected with furin siRNA exhibited higher ratio of apoptotic cells as compared to the control siRNA group (*, *P*<0.05). B. Hoechst staining of cells transfected with control siRNA or furin siRNA. Healthy cells showed round and intact nuclei, whereas apoptotic cells exhibited nuclear karyopyknosis or fragmentation as the arrows showed. The right four images are the high magnification of the framed region of the figures on the left. C. Expression of furin in the granulosa cells after siRNA transfection. The level of furin was significantly decreased after RNAi (**, *P*<0.01). α-tubulin was included as a loading control.

As furin was primarily expressed in the granulosa cells and furin is known to increase pro-transforming growth factor beta (pro-TGF β) leading to the production of bioactive TGFβ, which plays an important role in proliferation and apoptosis of granulose cells, we hypothesized furin might participate in the development of granulosa cells. We first evaluated the role of furin in the apoptosis of granulosa cells. Primary rat granulosa cells harvested from immature rats after 48 h of PMSG injection were transfected with furin siRNA or control siRNA for 48 h. Cells were then harvested and incubated with Hoechst 33258 staining buffer and the apoptotic cells were identified morphologically by fluorescent microscopy (Figure 3B). The percentage of apoptotic cells was significantly increased in the furin siRNA-transfected cells, as compared to the group transfected with the negative control siRNA (Figure 3A; *P*<0.05).

### Pro-apoptotic proteins were increased while anti-apoptotic proteins were decreased after furin was knocked-down

**Figure 4:**
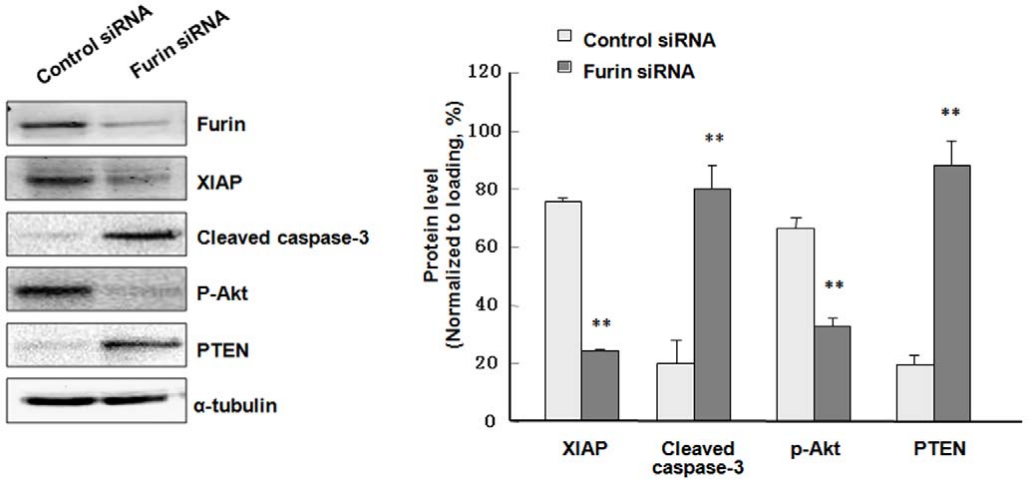
Western blot analysis of apoptotic-related proteins after furin siRNA transfection. Gramulosa cells were transfected with furin siRNA or control siRNA for 48 h before being subjected to protein extraction and Western blot with the indicated antibodies. Shown at the left were representative Western blot images. Three such experiments were quantified by measuring the intensity of apoptotic-related proteins relative to the α-tubulin (loading) control. The anti-apoptotic proteins XIAP and p-Akt were decreased by appropriate 3-folds (**, *P*<0.01) and 2-folds (**, *P*<0.01). On the contrary, the levels of pro-apoptotic, cleaved caspase-3 and PTEN were increased appropriate 4-folds (**, *P*<0.01) and 4.5-folds (**, *P*<0.01).

To further assess the role of furin in granulosa cell apoptosis, we next evaluated expression of apoptosis-related proteins. These include anti-apoptotic XIAP and p-Akt, and pro-apoptotic cleaved caspase-3 and PTEN. Western blot results and statistical analysis indicated that furin siRNA decreased the levels of XIAP by approximately 3-fold (*P*<0.01) and p-Akt by 2-fold (*P*<0.01). On the contrary, the levels of pro-apoptotic, cleaved caspase-3 and PTEN were increased appropriate 4-fold (*P*<0.01) and 4.5-fold (*P*<0.01), respectively, after furin siRNA transfection (Figure 4).

### Proliferation of granulosa cells was inhibited by furin siRNA in rats

**Figure 5:**
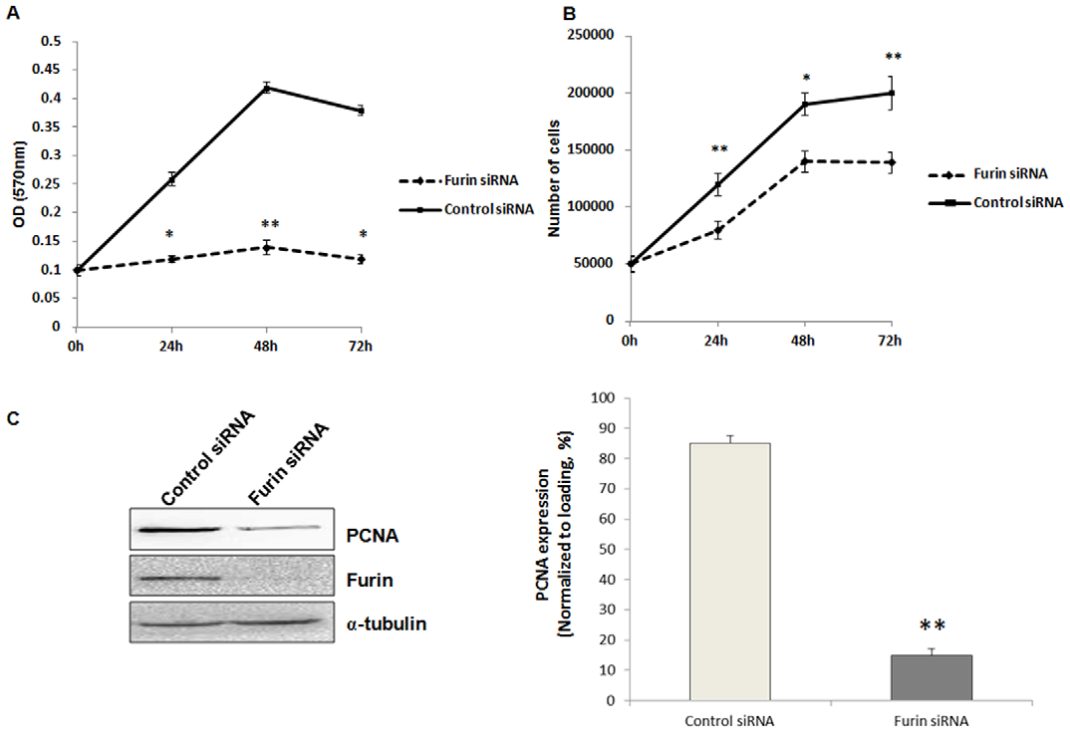
Proliferation of granulosa cells was inhibited by furin siRNA. Granulosa cells were transfected with furin siRNA or contron siRNA for 48 h before being included in the proliferation assay. A. Cell proliferation was examined by MTT assay. Data represented means ± SD of OD (570 nm) at 0, 24, 48, 72 h of siRNA transfection (each concentration was tested in triplicate). *, *P*<0.05 and **, *P*<0.01 were significantly different from control values evaluated by *t* test. B. Cell numbers after 0, 24, 48, 72 h of siRNA transfection. C. Western blot analysis of PCNA expression in granulosa cells after transfection with furin siRNA for 48 h. α-tubulin was used as a loading control. Shown on the left were representative Western blot images and on the right was the statistical analysis of three independent experiments (**, *P*<0.01).

Furin has been shown to be associated with enhanced invasion and proliferation in head and neck, breast, and lung cancers [17], [30], [31], [32]. To examine whether furin was also associated with proliferation of granulosa cells, granulosa cells from large antral follicles transfected with control siRNA or furin siRNA for 48 hours were subjected to MTT assay or counted by Scepter cell counter. The results showed that furin siRNA decreased proliferation of granulosa cells as compared to the control siRNA-treated group (*P*<0.05, Figure 5A, 5B). We further detected the expression of PCNA by Western blot, and PCNA was significantly decreased in the granulosa cells transfected with furin siRNA as compared to the control siRNA-treated cells (*P*<0.01, Figure 5C).

## Discussion

Many proteins are produced as proproteins and are activated by post-translational modification. The main function of PCSKs is to activate large numbers of proprotein substrates. PCSKs thus play essential roles in many processes such as the activation of proenzyme, synthesis of peptide hormones, processing of virus protein and maturation of receptor proteins. The main proprotein substrates include neuropeptide, polypeptide hormones, growth factors and their receptors and MMPs [14]. Furin is a cellular endoprotease that cleaves and activates many proprotein substrates. Furin has an essential role in embryogenesis and the maturation of proprotein substrates that range from growth factors and receptors to extracellular-matrix proteins and even other protease systems that control diseases [14].

PCSKs play important roles in the ovary. PCSK5 is highly expressed in the granulosa cells and theca cells of preovulatory follicles, and participates in cumulus expansion and follicular rupture by processing pro-TGFβ and pro-MMPs [33]. Furthermore, PCSK5 correlates with inhibin subunits in individual follicles in the mouse ovary [25], while PCSK4 is thought to participate in sperm capacitation and folliculogenesis [34], [35], [36]. PCSK6 is expressed in granulosa cells of small to medium preantral follicles but not in antral follicles [37], consistent with growth and differentiation factor 9 (GDF9) and Anti Mullerian Hormone (AMH) of the TGFβ superfamily [38], [39], [40], [41]. PCSK6 represents another granulosa cell transcript regulated by oocytes, but exhibits a novel expression pattern during follicular development [37]. Furin is also shown to participate in the process of ZPC secretion and ovulation [22], [23].

In the present study, we demonstrate that furin is involved in the development of granulosa cells during folliculogenesis. First, furin protein and mRNA were highly expressed in granulosa cells and oocytes (Figure 1 and 2). Antenos and colleagues [42] also found that activin could stimulate the expression of furin mRNA and the regulation of furin and inhibin subunits cooperated in a novel positive feedback loop that augments the regulation and secretion of bioactive mature activin-B and inhibin B dimers, necessary for local follicle stimulating hormone (FSH) β regulation. This indicates that furin plays an important role in regulation of hormone biosynthesis and secretion from pituitary gonadotrope cells. Additionally, it provides evidence that furin may be involved in the increased bioavailability of FSHβ mediators such as mature inhibin and activin dimers. FSH was important for the granulosa cells differentiation [6], [43]. Thus, we hypothesized that furin may participate in the development of granulosa cells.

Further investigation confirmed the role of furin in the development of granulosa cells based on the following lines of evidence: furin siRNA increased the apoptosis of granulosa cells by regulating the apoptosis-related factors XIAP and caspase-3, p-Akt and PTEN (Figure 3A, Figure 4). Meanwhile, the proliferation of granulosa cells was also inhibited (Figure 4A, 5B) by furin siRNA, and was associated with downregulation of PCNA (Figure 5C). The fate of granulosa cells determines the ability of follicles to continue to grow or undergo follicular atresia, and apoptosis of granulosa cells represents a major mechanism of the pathogenesis of follicular dysplasia [44]. As furin participates in ZPC secretion and ovulation, it also regulates the proliferation and apoptosis of granulosa cells and follicular development. Furin represents another granulosa cell-specific post-transcription regulation factor but exhibits a novel expression pattern during folliculogenesis.

Taken together, our data support a role of furin in regulating the apoptosis and proliferation of granulosa cells. These results may provide important implications for diseases such as POF or PCOS and infertility due to the abnormal development of granulosa cells. Further studies will be needed to define the exact mechanism by which furin regulates the apoptosis and proliferation of granulosa cells and the role of furin during folliculogenesis.
